# From lab to concert hall: effects of live performance on neural-acoustic phase-locking and engagement

**DOI:** 10.1093/scan/nsag021

**Published:** 2026-03-19

**Authors:** Arun Asthagiri, Psyche Loui

**Affiliations:** Department of Music, College of Arts, Media and Design, Northeastern University, Boston, MA 02115, United States; Department of Strings, New England Conservatory of Music, Boston, MA 02115, United States; Department of Music, College of Arts, Media and Design, Northeastern University, Boston, MA 02115, United States

**Keywords:** liveness, performance, music, entrainment, tracking, coupling, EEG

## Abstract

Live music performances continue to captivate audiences despite widespread availability of high-quality recordings, yet the neural mechanisms underlying the experience of live music remain poorly understood. This study investigates the effect of live versus recorded music on neural-acoustic phase-locking, an index of neural coupling with acoustic rhythms. Twenty-one participants listened to two live and two recorded performances of fast and slow movements of J.S. Bach’s works for the solo violin in a concert hall setting, while their EEG data were collected. Participants made behavioral ratings of engagement, spontaneity, pleasure, investment, focus, and distraction after each trial. Live performances were rated higher than recorded performances on a pleasure-engagement dimension. Live trials showed significantly higher neural-acoustic phase-locking than recorded trials in frequencies corresponding to rhythmic timescales within the excerpts. A follow-up analysis linked the effects of liveness on phase-locking to the increases in pleasure and engagement reported for live over recorded trials. Altogether, results demonstrate that live music strengthens dynamical responses to musical rhythm within the brain, which provides a candidate neural basis for the widespread appeal of live music and theories of social bonding through music.

## Introduction

Music is often cited as one of life’s greatest pleasures, and despite the proliferation of audio and video streaming services, attending live music performances remains one of the most popular activities across age groups and cultures. A growing body of literature shows that live, in-person experience plays an important role in social interaction, mental wellbeing, and collaborative communication (Balters *et al.* 2023, Stieger *et al.* 2023). Audiences cite a variety of psychological reasons for being drawn to live performances, including social engagement and novelty seeking (Brown and Knox 2017), though less is known about the neural dynamics underlying the perception of live music.

When listening to naturalistic music and speech, rhythms in the acoustic signal are reflected in low-frequency neural activity across distinct timescales in the delta (0.5–4 Hz) and theta (4–8 Hz) bands (Nozaradan *et al.* 2011, Doelling and Poeppel 2015, Donhauser and Baillet 2020). Nested timescales of neural–acoustic alignment are thought to represent hierarchical levels of contextual integration (Lakatos *et al.* 2005, Giraud and Poeppel 2012). During continuous speech, the brain tracks low-level syllabic units in the theta-band and high-level phrasal and sentential structure in the delta-band (Ding *et al.* 2016). During music listening, where periodicity in the acoustic signal is organized by musical rhythm (Harding *et al.* 2025), neural activity aligns with harmonics of the beat rate (Tichko *et al.* 2022). Theta activity tracks rhythms at the note rate, whereas delta activity underlies the perception of larger-scale groupings (i.e. the beat and metrical structure) (Nozaradan *et al.* 2011, Zalta *et al.* 2024). Consequently, nested timescales of neural activity within the delta- and theta-bands may parse multiple levels of musical structure.

Neural–acoustic alignment can be quantified in the phase domain through phase-locking (Lachaux *et al.* 1999), which measures cyclic coupling between neural and acoustic rhythms over distinct timescales. Neural–acoustic phase-locking occurs when patterns of neural activity consistently align with acoustic inputs at specific frequencies (Cohen 2014, Obleser and Kayser 2019). The question of how acoustic rhythms give rise to phase coherence in the brain is a point of ongoing discussion. Theoretical accounts posit that phase-locking can emerge from both transient evoked responses to acoustic events as well as entrainment of endogenous neuronal oscillations (Novembre and Iannetti 2018, Obleser and Kayser 2019, Duecker *et al.* 2024). The present study does not aim to disentangle the two, rather, we use phase-locking as a measure of neural sensitivity to musical rhythm across distinct timescales.

Phase-locking to rhythm facilitates auditory perception at multiple levels. At the level of acoustic parsing, the phase of ongoing neural activity tracks periodic modulations in the acoustic signal and influences detection accuracy for events in the auditory stream (Henry and Obleser 2012). Perceptual and neural fluctuations induced by acoustic rhythms continue past the point of rhythmic stimulation (Hickok *et al.* 2015, Zoefel *et al.* 2024, Henry *et al.* 2025), suggesting a causal relationship between the phase of neural activity and perceptual sensitivity under certain contexts, which might be attributed to shifts in neuronal excitability (Buzsáki and Draguhn 2004, *Lakatos et al.* 2008). The relationship between phase-locking and the fidelity of auditory perception extends to naturalistic settings, where, for instance, the intelligibility of spoken sentences is linked to phase-coherence in the theta-band (Luo and Poeppel 2007, Peelle *et al.* 2013). At the level of temporal expectations, paradigms that control for low-level acoustics suggest that cortical coupling in the delta- and theta-bands supports the anticipation of words and phrases (Ding *et al.* 2016, Kösem *et al.* 2018), as well as the expectation of the musical beat (Tal *et al.* 2017). Together, these empirical findings indicate that phase-locking, regardless of whether it is generated by an evoked or oscillatory process, reflects the brain’s ability to perceive and predict acoustics over multiple rhythmic timescales. As a result, neural–acoustic phase-locking provides insight into how dynamic neural responses to acoustic input contribute to perceptual engagement with live performance.

We posited that two key attributes of live performance might influence neural-acoustic phase-locking: the engagement with (awareness of and attention toward) a live performer, as well as acoustic variation between performances themselves. Studies in selective attention show that directing attention toward an auditory stream increases neural-acoustic phase alignment, which biases perception (Lakatos *et al.* 2008, Zion Golumbic *et al.* 2013). As a result, phase-locking is thought to be modulated by top-down (i.e. attentional) factors (Calderone *et al.* 2014, Lakatos *et al.* 2019). In music, these top-down factors may include the perceptual context of a rhythm (e.g. whether it is human-generated; Cameron *et al.* 2019), as well as acoustic features, such as spectral complexity (Wollman *et al.* 2020) and pitch contour (i.e. in song versus speech, Vanden Bosch Der Nederlanden *et al.* 2020). In the context of live performance, it follows that both higher-level factors like the perceptual awareness of a live performer, as well as lower-level features of the acoustics, might contribute to phase-locking and behavioral engagement.

Recent research into live performance reveals a link between interpersonal dynamics and neural engagement. For instance, altering audience-performer interactions through neural feedback increases activity within the auditory association cortex (i.e. posterior superior temporal gyrus) and subcortical limbic (i.e. ventral striatum and amygdala) regions (Trost *et al.* 2024). In addition, live over recorded performance enhances interpersonal neural synchrony in the delta-band amongst audience members (Rai *et al.* 2025). Our study addresses the mere perceptual and acoustic context of liveness at the individual level. To this end, we manipulate the awareness and experience of life relative to recorded music while minimizing differences in sensory input between the two. Specifically, this preregistered study uses EEG to test whether and how live performances compared to recorded controls affect neural–acoustic phase-locking in a concert hall. Although the preregistration included an additional hypothesis about EEG power, analyses related to that hypothesis are not reported here. We hypothesized that live over recorded music would (i) result in more positive reports of pleasure and engagement and (ii) increase the strength of neural–acoustic phase-locking to rhythms in the music.

## Methods

### Participants

Twenty-one participants (11 female, mean age 22.9 yrs, range 18–31 yrs) were recruited from online surveys and advertisements from the New England Conservatory of Music community. The preregistered sample size was determined based on prior published research on neural–acoustic phase-locking (Harding *et al.* 2019, Vanden Bosch Der Nederlanden *et al.* 2020) and related work from our lab (Przysinda *et al.* 2017) in accordance with scheduling limits for the concert hall. Though musical training was not an inclusion criterion, all participants who enrolled in the study had a history of formal musical training (n = 5: 3–9 yrs; n = 16: > 10 yrs) in violin, other strings, winds, or voice (n = 7, 8, 3, 3, respectively), likely due to the conservatory population from which we recruited. Participants gave informed consent as outlined in IRB #18-12-13 and were compensated $25.

### Stimuli

**Figure 1:**
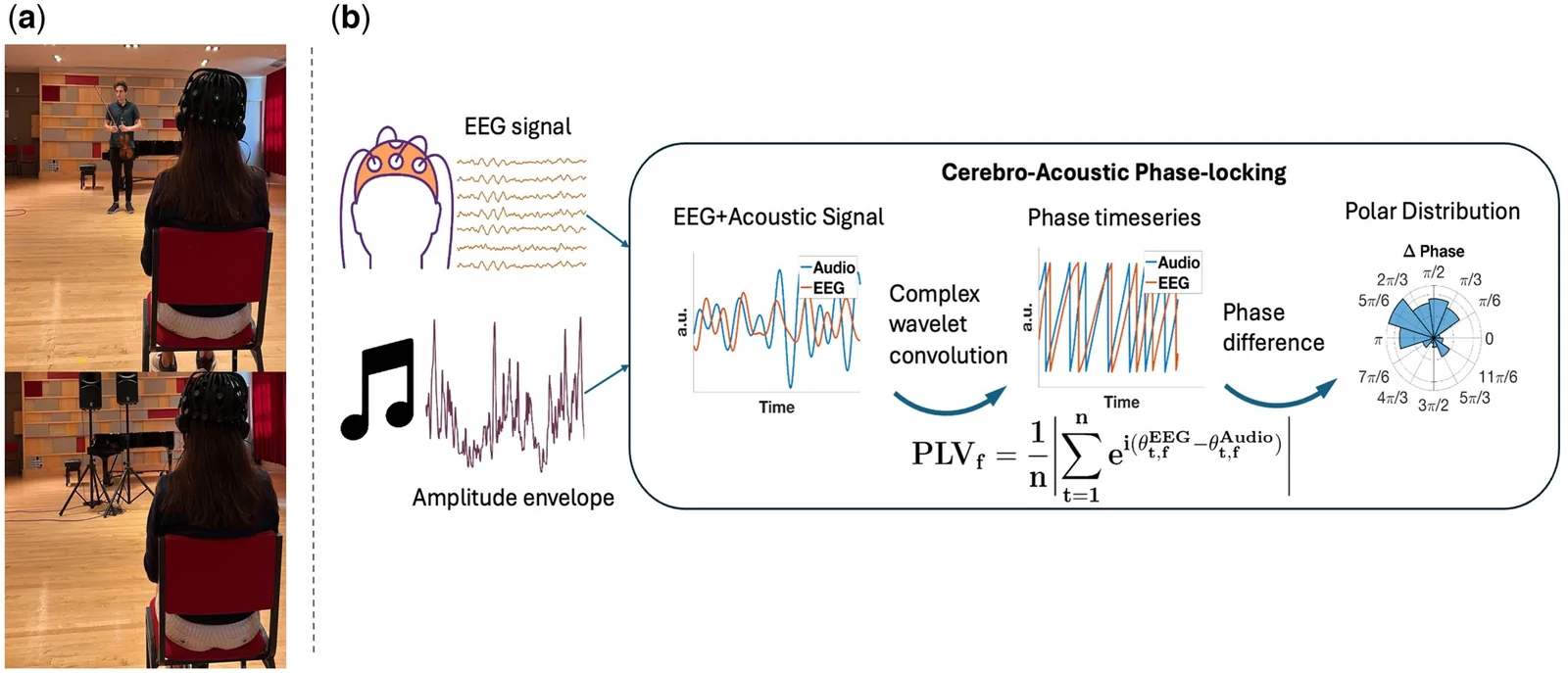
Data collection and analysis methods. (a) Live and recorded performances were presented separately to 21 participants in a concert hall (eyes closed). EEG was recorded using a mobile system. Live and recorded trials were matched for loudness and acoustic source location. (b) Phase-locking and the temporal response functions revealed naturalistic neural–acoustic phase-locking provided a measure of the phase-angle coherence between acoustic and EEG signals at a given frequency.

Four excerpts were selected from J.S. Bach’s Solo Violin Sonatas and Partitas as stimuli for the experiment. Two fast movements (approx. 125 beats per minute) and two slow movements (approx. 50 bpm) were selected to match for style and structure within tempo conditions: Sonata No. 1 mov. 1—Adagio (slow), Sonata No. 2 mov. 1—Grave (slow), Partita No. 1 mov. 1—Preludio (fast), and Sonata No. 3 mov. 4—Allegro (fast). Two of these movements, one fast and one slow, were presented in live performance by the renowned violinist Joshua Brown. The other two movements were presented with a dual-speaker system (see Fig. 1a). The order of live and recorded stimuli was alternated within each participant and counterbalanced across participants. The order of tempo was pseudorandomized across participants such that every participant experienced all four Bach excerpts once each, and every participant experienced four conditions in a 2 × 2 design: Live-Fast, Live-Slow, Recorded-Fast, and Recorded-Slow. Each excerpt was randomly assigned to the live or recorded condition per participant within the tempo constraints. Thus, each participant experienced all four conditions in the 2 x 2 design.

### Procedure

Participants listened to four excerpts (live/recorded × fast/slow) presented in a concert hall (Pierce Hall) at the New England Conservatory. EEG was recorded with eyes closed to minimize visual differences between the performer’s movements and the static speaker. This reduced the difference in overall stimulus intensity between live and recorded performance. Before live trials started, participants were able to observe the live performer (or the speaker) on stage. Thus, our manipulation emphasized the awareness and experience of live relative to recorded music and minimized the difference in the magnitude of sensory input between the two. The two live performances per participant were given by concert violinist and soloist, Joshua Brown, who played on a 1679 Guarneri Del Gesu violin. The tempo of each excerpt was played on a metronome for the violinist before performances to ensure consistency of tempi across trials. The two recorded excerpts were played from recordings made by the same violinist in the same concert hall prior to data collection.

After informed consent procedures, participants completed questionnaires on demographic information and standardized assessments of musical reward sensitivity (Extended Barcelona Music Reward Questionnaire, eBMRQ, (Mas-Herrero *et al.* 2013, Cardona *et al.* 2022) musical sophistication and engagement (the Goldsmiths Music Sophistication Index, Gold-MSI, Müllensiefen *et al.* 2014), The Synesthesia Battery (Eagleman *et al.* 2007), and the Adult ADHD Self-Report Scale (Kessler *et al.* 2005). Directly after each trial, participants completed survey questions about their listening experience (Figure S1, see online supplementary material for a color version of this figure). The experiment generally lasted 1 hour for each participant including EEG setup, questionnaires, and listening.

### Data acquisition

EEG data were recorded using a dry Quick-32r headset at 500 Hz and collected with the CGX custom acquisition software. Electrode impedances were kept under 300 kOhms. A CGX Wireless Stim Trigger device was used to align audio recordings with EEG data at the start of each trial. All live and recorded performances were recorded using Pierce Hall’s DPA 4011C stereo microphone system. For two excerpts, backup recordings from a separate Blue Yeti microphone were used due to errors with the recording system.

A Yamaha 600S dual-speaker system presented recorded excerpts for the first 11 participants, and a JBL Eon 500 (a comparable PA dual-speaker system) presented recordings for the remaining 10 participants. The playback system and performer was set 14 feet from the participant (measuring audio source to ear), the bottom of the speakers was situated 4.5 feet above the ground to match the height of the violin, and the dual speakers were placed close together (approx. 1 ft 10 inches apart) to simulate the single sound source of the violin. We matched the volume of the speaker and acoustic violinist prior to data collection by recording the violinist and the speaker separately for the same clip (opening of the Bach A Minor excerpt) and analyzing the audio levels offline. We iteratively adjusted speaker levels until there were minimal differences in overall loudness between the acoustic violin and speaker. We performed a post-hoc analysis on raw recordings to confirm that loudness did not differ between live and recorded trials (see “Data Acquisition” in Supplementary Methods).

## Analysis plan

### Behavioral data analysis

The behavioral analysis tested the preregistered hypothesis (hypothesis 1) that participants would rate live over recorded music higher on multiple factors of audience engagement. After each excerpt, participants gave 5-point Likert ratings on multiple dimensions of emotional engagement (Figure S1, see online supplementary material for a color version of this figure). We opted for a data-driven approach to reduce the dimensionality of the questionnaire responses without imposing a selection bias for which items to retain. Since questionnaire items covered related constructs (i.e. engagement and investment), we expected latent sources of variability to underlie participant responses. To elucidate these shared sources of variance across items and reduce the dimensionality of the data, we used principal component analysis (PCA) (princomp function in R).

The first and second principal components explained 48.3% and 16.2% of the variance in the data, respectively. We did not retain additional principal components because the explained variance tapered after the second principal component (Figure S2A, see online supplementary material for a color version of this figure). Projections of participant ratings onto the first principal component were defined as “pleasure-engagement” scores, and projections onto the second principal component were defined as “distraction-familiarity” scores, reflecting relative contributions of questionnaire items (Figure S3, see online supplementary material for a color version of this figure). Since we hypothesized that positive engagement would vary as a function of liveness, our statistical analysis focused on pleasure-engagement scores (PC1). We entered pleasure-engagement scores into a generalized linear mixed-effects model (glmmTMB in R) to test for tempo, liveness, and the tempo: liveness interaction as fixed-effects, with participant as a random intercept.


PC1∼livesness*tempo+(1|sub.idx)

### Audio preprocessing

Stereo recordings were converted into mono tracks by averaging across channels for subsequent analyses. Recordings from each trial were also normalized for loudness using Matlab’s Audio Toolbox (v. 24.1) to obtain a final gain of −20 LUFS. Audio normalization was performed to account for potential changes in the concert hall microphone levels, which may have varied slightly between participant sessions due to concert hall use outside of this study. To justify normalization, we tested for loudness differences between all unnormalized live and recorded excerpts and re-ran the acoustic follow-up analysis with unnormalized audio (see “Audio Preprocessing” in Supplementary Methods).

Additional acoustic preprocessing steps were carried out for the phase-locking analysis using MIR Toolbox (v. 1.8.2, Lartillot *et al.* 2008). Normalized audio was filtered across 50 logarithmically spaced frequencies using a gammatone filterbank (mirfilterbank) to simulate the filtering of the cochlea in the inner ear, the amplitude envelope was computed within each frequency band using a lowpass FIR filter (mirenvelope) and summed across frequency bands (mirsum) to obtain a single-channel acoustic envelope signal. This signal was downsampled to 500 Hz to match the EEG sampling rate.

### EEG preprocessing

The EEG was preprocessed using a custom pipeline with EEGLab functions (Delorme and Makeig 2004). A detailed report of EEG preprocessing steps is given in the Supplementary Methods. Data was referenced to the right earlobe (A2) and bandpass filtered between 0.2 and 58 Hz. Noisy channels were automatically identified and rejected based on flatline criteria (>0.5 seconds) and correlations with neighboring channels (r < 0.5). Artifact Subspace Reconstruction (ASR) was used to reconstruct noisy data epochs from calibration data. Noisy channels were spherically interpolated. Finally, Independent Component Analysis (ICA) was used to remove independent spatial components of the data that reflected signal artifacts.

### Neural–acoustic Phase-Locking analyses

We tested the preregistered hypothesis (hypothesis 2) that live over recorded music would result in stronger neural–acoustic phase-locking at distinct rhythmic timescales in the music. Neural–acoustic phase-locking reveals cycling coupling between neural and acoustic signals, which we operationalize through the phase-locking value (Lachaux *et al.* 1999) (Fig. 1b). We were particularly interested in phase-locking at harmonics of the beat rate, which typically fall within the delta (0.5–4 Hz) and theta (4–8 Hz) frequency ranges (Ding *et al.* 2017). Previous literature also links rhythmic expectation in music to the beta (15–30 Hz) range (Doelling and Poeppel 2015). Thus, we measured phase-locking across a broad range of frequencies ranging from the delta to beta bands (0.2–20.2 Hz) and corrected for multiple comparisons across frequency bins using FDR correction.

Using complex Morlet wavelet convolution, we obtained an analytic signal for audio and EEG at frequencies linearly spaced from 0.2 to 20.2 Hz with a frequency increment of 0.2 Hz. A wavelet of five cycles was selected, which is consistent with three to seven cycles typically used in the phase-locking literature (Harding *et al.* 2019, Wollman *et al.* 2020, Tichko *et al.* 2022). After wavelet convolution, we calculated the time-resolved phase difference between the resulting EEG and acoustic analytic signals. Finally, we derive the phase-locking value from the phase difference series by taking the magnitude of the mean complex vector (see equation below). A single phase-locking value was calculated per frequency, electrode, and trial (Lachaux *et al.* 1999).


$${\text{PLV}}_{f}=\left|\frac{1}{n}\sum_{t=1}^{n}e^{i(\theta_{t,f}^{\mathrm{EEG}}-\theta_{t,f}^{\mathrm{Audio}})}\right|$$

Phase-locking values were averaged across the three frontocentral electrodes (F3, Fz, F4), which elicited the highest coherence and aligned with the acoustic phase-locking literature (Harding *et al.* 2019, Vanden Bosch Der Nederlanden *et al.* 2020, Wollman *et al.* 2020). We fit generalized linear mixed effects models (log-link function) separately for fast and slow excerpts to test effects of live versus recorded performances on phase-locking strength, accounting for variance between excerpts and participants (model formula below). The model was fit separately for each frequency, and *P*-values were corrected for multiple comparisons using Benjamini & Hochberg FDR. Finally, we performed a confirmatory analysis using the same model on phase-locking values averaged across the significant contiguous frequency range for the fast excerpts.


$${\mathrm{PLV}}_{f,\mathrm{tempo}}\;∼\mathrm{livesness}+\mathrm{excerpt}+(1|\mathrm{sub}.\mathrm{idx})$$

### Brain-behavior relationships

As an exploratory analysis, we assessed relationships between the strength of phase-locking and the perceived effect of liveness as operationalized by the behavioral ratings. We related the within-subject difference between the average phase-locking value over significant frequencies in the fast excerpts (the outcome variable in the confirmatory PLV analysis) to the within-subject difference in pleasure-engagement scores (the outcome variable in the behavioral analysis), using a generalized linear model. $\left[PLV_{\mathrm{live}}-{\mathrm{PLV}]}_{\mathrm{recorded}}∼[\mathrm{BehavioralScor}e_{\mathrm{live}}-\mathrm{BehavioralScor}e_{\mathrm{recorded}}\right]$ (No random effects were included at the participant level because the regressor and outcome variables were derived from within-participant differences).

### Acoustic follow-up analyses

We performed a second exploratory analysis to investigate whether low-level acoustic features impacted neural–acoustic phase-locking and pleasure-engagement over and above the effects of liveness. Using MIRToolbox (Lartillot *et al.* 2008), we extracted six acoustic features from the recordings of each trial: three features characterized changes in the time domain, i.e. “temporal features”: RMS loudness (mirrms), pulse clarity (mirpulseclarity), and spectral flux (mirflux) and three features characterized timbral components, i.e. “spectral features”: spectral centroid (mircentroid), brightness (mirbrightness), and zero-crossings (mirzerocrossings). We used Principal Component Analysis (princomp in R) to reduce the dimensionality of these acoustic descriptors and applied factor rotation (varimax in R) to potentially highlight differences between tempo and liveness conditions. The first and second principal components explained 48.6% and 39.6% of the variance in the data, respectively. We did not retain additional principal components because the explained variance dropped steeply and tapered after the second principal component (Figure S2B, see online supplementary material for a color version of this figure).

We assessed whether the first principal component (which differentiated live and recorded conditions) contributed to behavioral and neural outcomes. We tested for collinearity between the acoustic first principal component and liveness in the phase-locking GLMM model (see “Neural–Acoustic Phase-Locking Analyses”). Due to multicollinearity (variance inflation factor = 13.85), we residualized the first principal component of the acoustics with respect to the liveness condition: we fit a linear model to predict the acoustic PC1 from the liveness condition (using the acoustic data from all live and recorded performances) and subtracted the predicted acoustics from the true acoustics to obtain the residualized acoustic PC1. We included the residualized acoustic PC1 as a regressor in the previous behavioral and phase-locking generalized mixed effects models (see “Behavioral data analysis” and “Neural–Acoustic Phase-Locking Analyses”) to test the hypothesis that acoustic variability beyond condition effects of liveness impacted behavioral and neural outcomes.

## Results

### Pleasure and engagement are higher during live music listening

**Figure 2:**
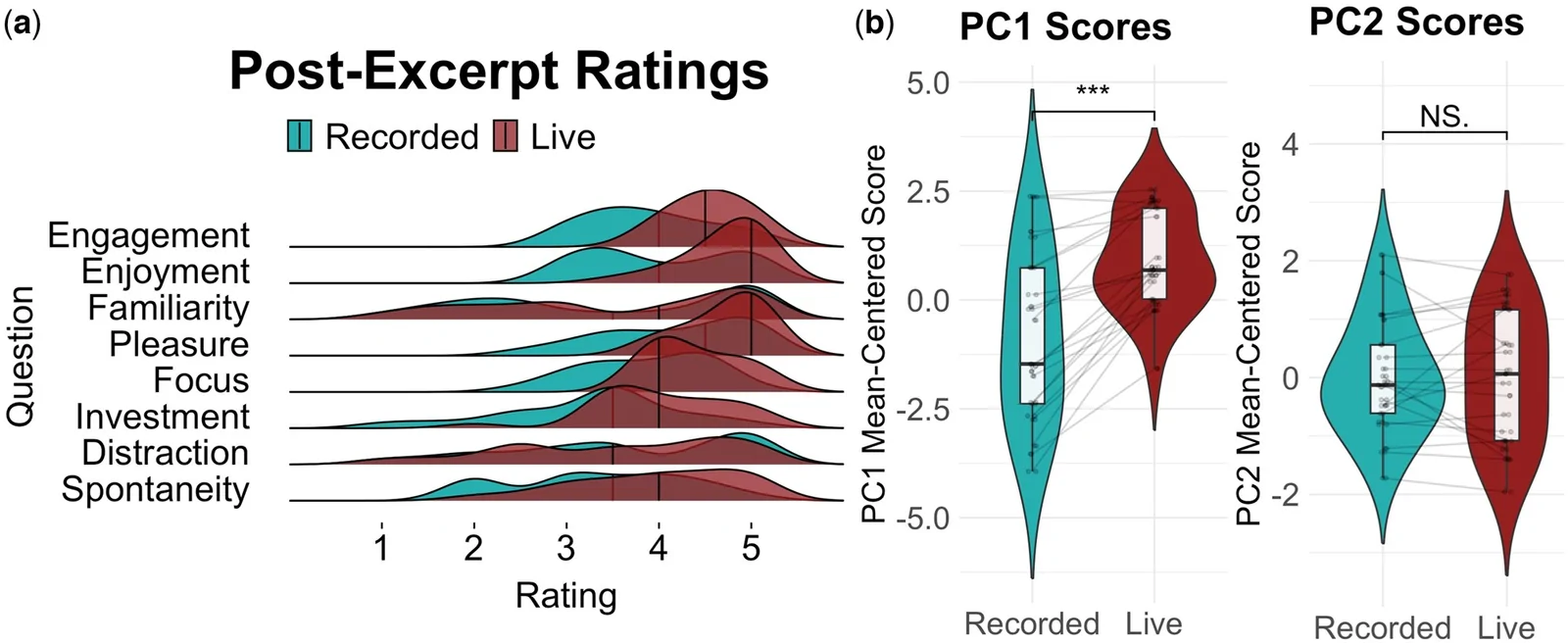
Behavioral responses to post-excerpt questionnaires. (a) Live compared to recorded trials were rated higher on engagement, enjoyment, pleasure, investment, and spontaneity, and similarly for familiarity and distraction. (b) The first principal component, which summarized a single “pleasure-engagement” dimension, was significantly higher for live over recorded performances (β = 0.25, SE = 0.046, z = 5.51, P<.001). The second principal component summarized a “distraction-familiarity” dimension and did not differ significantly between live and recorded performances.

Our first hypothesis (preregistered) was that live music would elicit more positive behavioral ratings than recorded music (see “Behavioral Data Analysis” under Methods). Indeed, participants generally rated live trials higher than recorded trials in engagement, enjoyment, pleasure, focus, investment, and spontaneity (Fig. 2a). Ratings of familiarity and distraction were similar between live and recorded performances. A principal component analysis (PCA) was conducted to examine shared sources of variance in survey responses (see Behavioral Data Analysis). This PCA revealed two principal components, defined as “pleasure-engagement” (PC1) and “distraction-familiarity” (PC2) (Figure S3, see online supplementary material for a color version of this figure). We assessed effects of liveness and tempo on pleasure-engagement scores using generalized mixed-effects models. Live music elicited significantly higher levels of pleasure-engagement than recorded music (*β = 0.25, SE = 0.046, z = 5.51, P<.001*, Table S1, see online supplementary material for a color version of this table) with a non-significant tempo interaction term (*β = −0.039, SE = 0.065, z = −0.60, P = . 55*) and a non-significant main effect of tempo (*β = 0.062, SE = 0.046, z = 1.34, P = .18*). The significant positive beta estimate for only liveness suggests that regardless of tempo, live music elicited a more positive subjective experience than recorded music.

### Liveness is linked to stronger phase-locking at salient acoustic frequencies

**Figure 3:**
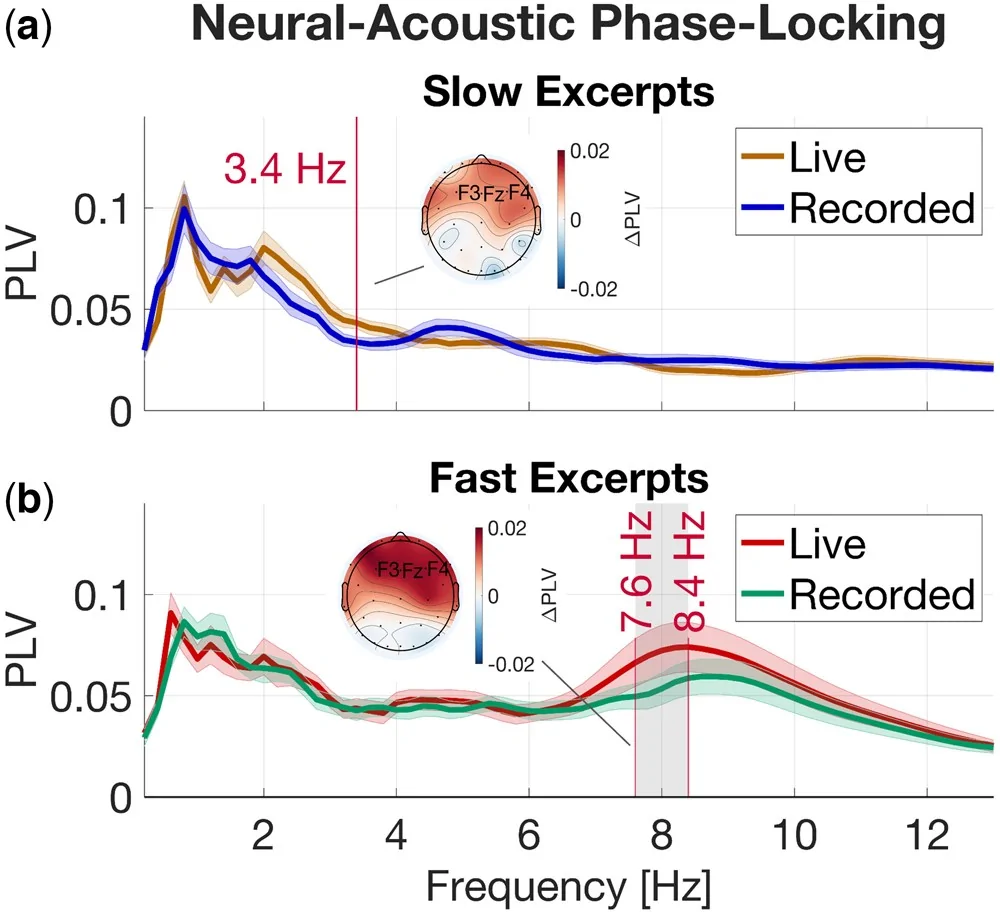
Cerebro-acoustic phase-locking differs across tempo and liveness. Phase-locking values between neural and acoustic activity plotted across a range of frequencies for (a) slow and (b) fast excerpts (shaded regions are ±1 standard error). Frequency ranges demarcated by vertical lines contained significant differences in phase-locking between live and recorded performances (generalized mixed effects models; FDR corrected *P* < .05). Topo plots show average differences in phase-locking strength over significant frequencies for live over recorded performances.

Next, we hypothesized (preregistered) that neural–acoustic phase-locking would be stronger for live over recorded music, and that the affected frequencies would be linked to the beat-rate of the music. We computed phase-locking values between cochlear-filtered acoustic envelope and EEG activity over a range of low (<20 Hz) frequencies to test for differences between conditions. Peaks in phase-locking occurred in the delta range for slow excerpts (Fig. 3a) and the theta range for fast excerpts (Fig. 3b), reflecting delta and theta rhythms in the acoustics themselves (Figure S2, see online supplementary material for a color version of this figure). The delta range (.5–4 Hz) included the beat rate and note rate of the slow excerpts (beat rate: 50 bpm = .83 Hz; note rate: 4*50 bpm = 3.33 Hz), whereas the upper-theta range included the note rate of the fast excerpts (note rate: 4*125 bpm = ∼8.3 Hz).

We tested for differences between live and recorded conditions using generalized linear mixed effects models at each frequency and corrected for multiple comparisons across frequencies using FDR. The model included liveness and excerpt as fixed effects and participant as a random effect. Live conditions showed significantly greater phase-locking than recorded conditions in the delta range (3.4 Hz) for the slow excerpts and in the upper-theta frequency range (7.6–8.4 Hz) for the fast excerpts (*P<.05, FDR corrected*). Frequencies with significantly higher phase-locking during live over-recorded performance also corresponded to the note rates of the slow and fast excerpts (3.33 Hz and 8.3 Hz, respectively).

We performed a confirmatory test on the average phase-locking value across this significant frequency range for the fast excerpts using the same model design. There was a significant effect of both excerpt and liveness on the strength of neural–acoustic phase-locking (Liveness: *β = 0.27, SE = 0.08, z = 3.36, P<.001;* Excerpt: *β = 0.44, SE = 0.08, z = 5.42, P<.001*, Table S2A, see online supplementary material for a color version of this table). The beta estimate for liveness represents a within-participant conditional effect on a log-transformed scale (due to the log-link function). To interpret the beta estimate in the original scale, we applied the inverse link function (the natural exponent) to the beta coefficient, which yields the ratio of the expected phase-locking between conditions. The model estimate revealed that the expected phase-locking value for live compared to recorded performance (within-participant) was 31% higher (e^0.27 = 1.31). The beta estimate for the excerpt effect indicates that the expected phase-locking value was 55% higher (e^0.44 = 1.55) when listening to the E Major Partita excerpt relative to the C Major Sonata excerpt. The excerpt effect might be attributed to compositional differences between excerpts (i.e. melodic contour and timbral register). Subsequent brain–behavior analyses focused on phase-locking over this upper-theta frequency range (7.6–8.4 Hz) of the fast excerpts as it showed a robust peak in phase-locking that aligned with the note-rate of these excerpts and significantly differed between live and recorded conditions.

### Individual differences in neural–acoustic phase-locking to live music relate to perceived pleasure-engagement

Given neural differences in phase-locking as well as behavioral differences in pleasure-engagement between live and recorded performances, we performed a follow-up analysis to examine whether phase-locking during music listening was linked to behavioral ratings post-listening. We conducted this exploratory analysis because we hypothesized that stronger neural–acoustic phase-locking during live over recorded performance might underlie the significant increase in pleasure-engagement ratings we had observed in the behavioral analysis.

**Figure 4:**
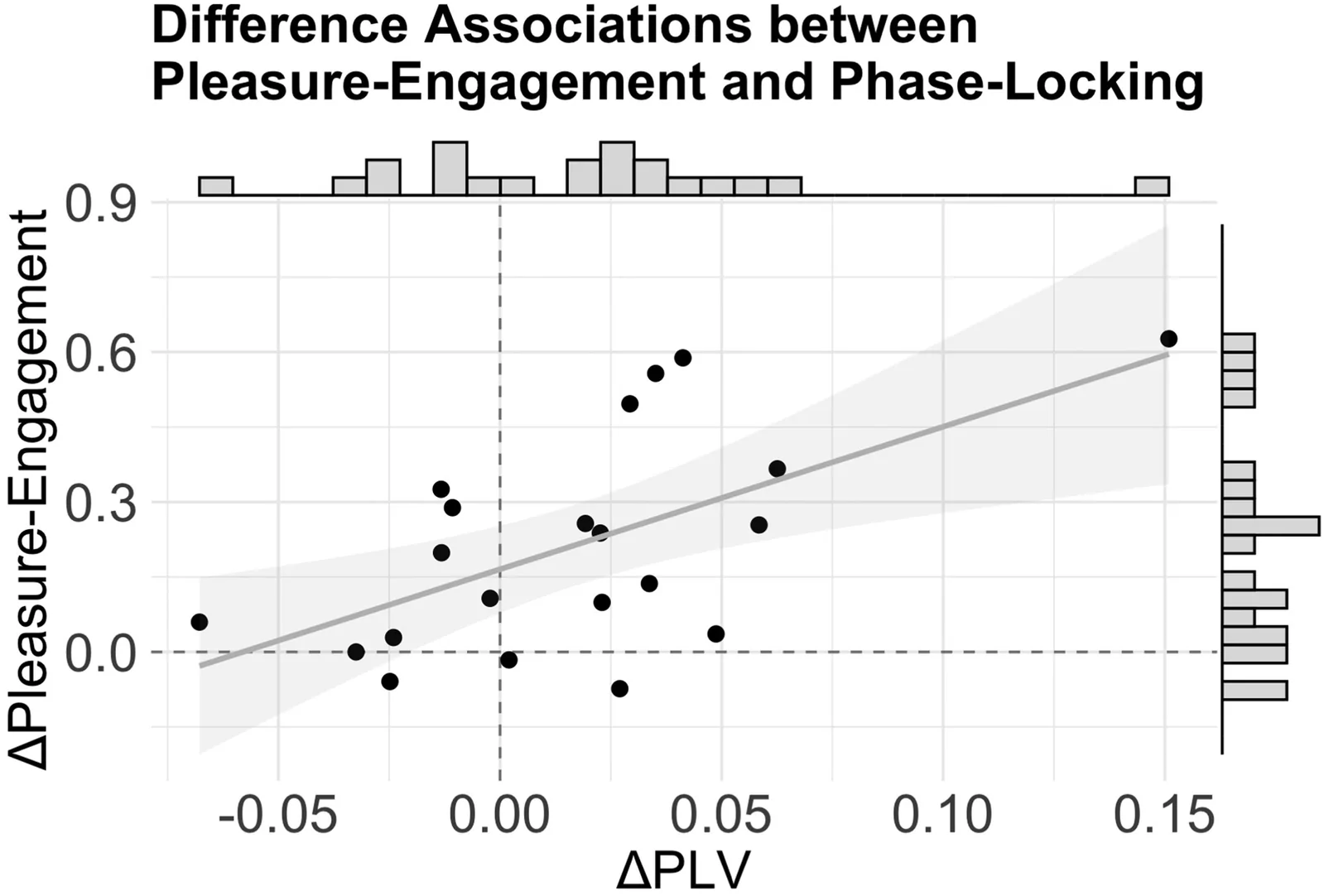
Phase-locking differences between live and recorded music predict differences in pleasure-engagement ratings. There was a significant relationship between the difference in phase-locking strength and the difference in pleasure-engagement ratings between live and recorded performances for fast excerpts (*P* < .001). Differences in pleasure-engagement (PC1) scores versus average phase-locking values between live and recorded performances are plotted. Points on the scatter plot denote single participants. Histograms show the marginal distributions of rating and PLV differences.

We tested whether mean phase-locking differences between live and recorded performances across the significant frequency range for the fast excerpts (7.6–8.4 Hz) predicted pleasure-engagement rating differences between the same trials. Results from a generalized mixed effects model revealed that phase-locking differences were significantly associated with pleasure-engagement differences (*β = 2.85, SE=.84, z = 3.40, P<.001*, Table S2B, see online supplementary material for a color version of this table). Specifically, stronger phase-locking during live relative to recorded music listening was significantly associated with higher perceived pleasure-engagement after listening (Fig. 4).

### Acoustic variability does not explain behavioral and neural outcomes beyond the manipulation of liveness

**Figure 5:**
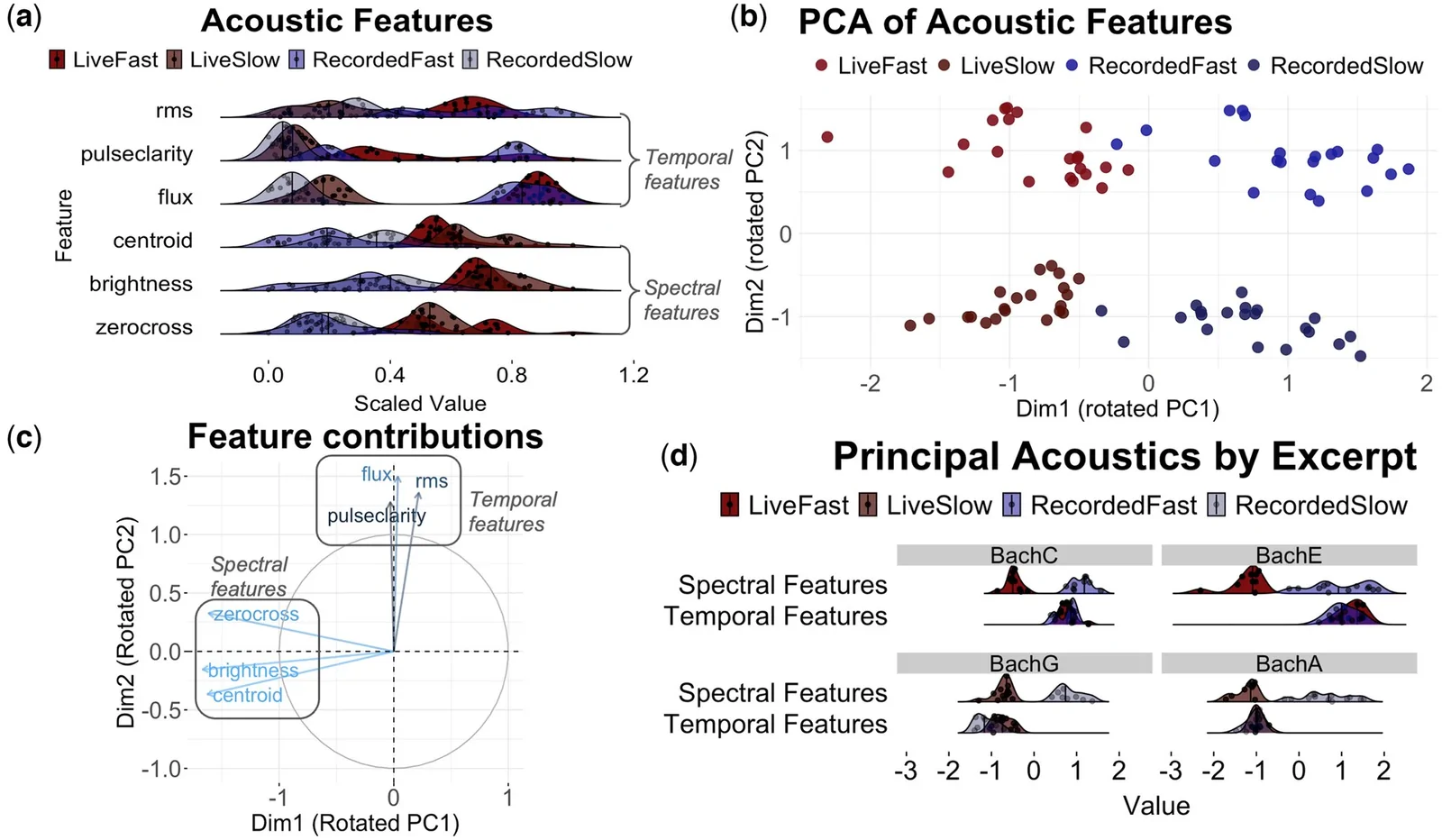
Acoustic features of live and recorded performance. (a) Density plots of acoustic features over trials show acoustic features that (b) distinguish liveness and tempo conditions after PCA. (c) Relative loadings reveal orthogonalized contributions of spectral (centroid, brightness, zero crossings) and temporal (RMS, spectral flux, pulse clarity) features. (d) Rotated PCs plotted within each excerpt highlight how spectral features differentiates liveness.

Since neural–acoustic phase-locking may be sensitive to both acoustic and perceptual features of natural listening, we performed an exploratory analysis to investigate whether variation in low-level acoustic features between performances impacted neural and behavioral outcomes. While our study did not separately manipulate top-down perceptual bias and bottom-up sensory input, we did record all live and recorded performances and were thus able to perform follow-up analyses to test for contributions from any salient acoustic features. We extracted low-level signal features from the normalized audio at the excerpt level, which included both spectral features (spectral centroid, brightness, zero-crossings) and temporal features (RMS energy, pulse clarity, and spectral flux) (Fig. 5a). Each of these features was summarized across an excerpt as a single value. These features were then inputted into a principal component analysis to reveal latent sources of acoustic variance (Fig. 5b).

We found that the rotated PC1 and rotated PC2 had distinct contributions from spectral and temporal features, respectively (Fig. 5c). The spectral dimension (PC1) differentiated between liveness conditions, whereas the temporal dimension (PC2) differentiated between tempo conditions (Fig. 5b and d). The spectral dimension (PC1) was sensitive to timbral differences between the acoustic violin and the speaker, which would have been apparent to listeners even after controlling for overall amplitude. The variance in PC1 scores was not larger for live than for recorded trials (variance across live trials: 0.033; variance across recorded trials: 0.074), suggesting that acoustic characteristics of live performances varied minimally across trials.

We examined whether acoustic PC1, which captured variance between live and recorded performances, influenced behavioral and neural outcome variables. Since the acoustic PC1 was strongly collinear with the liveness condition, we extracted the portion of acoustic variance that extended beyond the experimental manipulation of liveness—we residualized the acoustic PC1 with respect to the liveness condition. In this way, we isolated natural acoustic fluctuation across live and recorded trials apart from the salient timbral difference between the conditions (i.e. the timbre of the acoustic violin versus the speaker).

We entered the residuals of the first principal component of the acoustics into the generalized mixed effects models used for the previous analyses. Acoustic residuals were not significant predictors for pleasure-engagement ratings (Acoustic residuals: *β = −0.05, SE = 0.042, z = −1.20, P = .23*, Liveness: *β = 0.26, SE = 0.046, z = 5.68, P<.001;* Tempo: *β = 0.08, SE = 0.05, z = 1.64, P = .10*, Liveness: Tempo: *β = −0.049, SE = 0.06t5, z = −0.76, P = .45*, Table S3A, see online supplementary material for a color version of this table) or phase-locking values (Acoustic residuals: *β = 0.12, SE = 0.15, z = 0.80, P = .43*, Liveness: *β = 0.26, SE = 0.08, z = 3.38, P < .001;* Excerpt: *β = 0.49, SE = 0.10, z = 4.77, P < .001*, Table S3B, see online supplementary material for a color version of this table). Together, these results suggest that acoustic variation between live and recorded performances, over and above the manipulated aspects of liveness, did not significantly explain differences in pleasure-engagement and neural–acoustic phase-locking.

## Discussion

We provide a novel account of the effects of live music on neural–acoustic phase-locking. We demonstrated that (i) listeners rated live music higher than recorded music along a pleasure-engagement dimension and that (ii) phase-locking at rhythmic frequencies was stronger for live over recorded performance. Together, our results point to the notion that the awareness and experience of a live performer enhance neural sensitivity to musical rhythm and contribute to the perceived pleasure and engagement of live music.

The concert hall setting for the experiment echoes a broad movement in cognitive neuroscience that uses ecologically valid paradigms to investigate the brain in its natural environment (Zaki and Ochsner 2009, Nastase *et al.* 2020). Given that liveness is inseparable from the cultural and societal expectations held by the audience (Auslander 2022), the concert hall setting was apt for this study. This setting set an implicit expectation that musical performances were genuine live performances, which was particularly important given that our manipulation was largely contextual—listeners may be more attuned to their perception of liveness in a concert hall rather than in a lab setting.

Complementing our naturalistic setup, we carefully controlled aspects of the sensory and perceptual environment. For instance, participants listened to the live performances individually, which narrowed the social factor to the perception of the performer. We also matched the amplitude of sensory input between live and recorded trials at both visual and auditory levels (see “Procedure” and “Data Acquisition”). Consequently, our results shed light on how the acoustic and perceptual contexts of liveness shape an individual’s experience of music.

### Live music increases phase-locking to musical rhythm

Neural–acoustic phase-locking during live and recorded performances emerged across rhythmic delta and theta frequencies. Slow excerpts showed peaks in the delta range (.5–4 Hz), which may indicate neural tracking of the larger-scale musical structure (i.e. harmonic transitions between chords as opposed to melodic embellishments). Fast excerpts showed an additional prominent peak in the upper-theta (∼8 Hz) range, which included the note rate. Theta phase-locking in the fast excerpts suggests a lower-level auditory process, potentially linked to encoding of individual notes from the acoustic stream, consistent with the hierarchical nature of rhythmic tracking (Ding *et al.* 2016, Donhauser and Baillet 2020, Tichko *et al.* 2022). While other studies additionally report engagement of the beta-band in predictive timing and motor responses to the musical beat (Doelling and Poeppel 2015, Fujioka *et al.* 2015, Zalta *et al.* 2024), we speculate that the lack of beta-band engagement in our experiment may be due to lower urges to move to the acoustic violin music. In addition, our phase-locking method focused on acoustic-brain relationships, which may not be sensitive to intrinsic auditory-motor dynamics in the beta-band (Fujioka *et al.* 2015, Large *et al.* 2015).

Critically, phase-locking was stronger for live over recorded music at rhythmic frequencies (i.e. the note rate of fast excerpts). This suggests that the context of live performance shapes neural responses to musical rhythms—more precisely, that liveness increases the consistency with which neural activity cyclically aligns with periodic acoustic events. Since our account does not distinguish entrained neural oscillations from evoked activity, we interpret our findings in terms of “neural entrainment in the broad sense,” drawing from Obleser and Kayser (2019). We do not claim the existence of endogenous neural oscillation. Instead, our results suggest that neural activity within the delta- and theta-bands is more sensitive to acoustic rhythms during live over recorded performance. This heightened neural sensitivity, as reflected through increased delta and theta phase-locking, may contribute to stronger pleasure and engagement experienced during live performance.

Our experiment also sheds light on the qualities of live performance that shape neural and behavioral dynamics. The results extend findings from Trost *et al.* (2024), who focused on the effects of neural feedback between a live performer and listener. Our study shows that the context of the live performer and the quality of their music are sufficient to foster stronger engagement and neural coherence even without explicit feedback. The temporal precision of EEG allowed us to trace the effects of liveness to responses to musical rhythm within the delta- and theta-bands. Given the role of rhythm in social bonding (Cirelli *et al.* 2018, Grahn *et al.* 2021), the results suggest that liveness itself may promote the social synchrony and group bonding observed in live musical settings.

### Individual differences in phase-locking relate to ratings of pleasure and engagement

Neural–acoustic phase-locking is typically considered a low-level auditory phenomenon, but the present findings show that it interacts with the perceived pleasure of acoustic stimuli in the context of live music. Previous work has linked perceived pleasure to inter-subject theta coherence during live performance (Chabin *et al.* 2022). Our data corroborates these findings by relating the pleasure of live performance to individual differences in neural phase-locking. This link might stem from the altered context of live performance. For instance, a shift in attentional engagement encouraged by the context of liveness might selectively enhance neural tracking of the acoustics via phase-alignment (Lakatos *et al.* 2008, Calderone *et al.* 2014). Top-down effects of attention on neural–acoustic phase-locking could potentially explain a more salient and positive experience of the live performance. Further work is needed to understand whether the top-down effects of perceptual context are indeed separate from bottom-up effects from the acoustics. Though our acoustic analysis considered low-level acoustic variance across performances, more granular aspects such as tempo variability within live performances were not considered in this study.

Finally, individual differences may influence the effects of liveness. For example, familiarity with the performer varied across our sample (Little to no acquaintance: n = 9, Somewhat acquainted: n = 9, Very well acquainted: n = 3), which could act as a moderating factor on liveness (though it would not impact the direction of the effect). Our sample was also highly musically trained, which has been shown to heighten acoustic phase-coherence (Doelling and Poeppel 2015, Harding *et al.* 2019), though our within-subjects design minimizes this confound. Still, musically experienced participants may be generally more sensitive to the subtle features of liveness, which means that effects of liveness may differ in a general population. Finally, future work may test contributions of musical reward sensitivity (i.e. measured with the eBMRQ) on neural and behavioral responses to live music, given the link between phase-locking and pleasure-engagement.

## Conclusion

Together, the study provides novel insights into the effects of live music on neural responses to musical rhythm in a controlled yet naturalistic concert hall setting. Employing phase-based methods for relating continuous EEG and acoustic signals, our results suggest that live over recorded music enhances neural–acoustic phase-locking to music, and that this change correlates with the perceived pleasure and engagement of listeners. Our results provide foundational context for the growing number of studies that examine complex neural and physiological dynamics during live concerts (Czepiel *et al.* 2021, Tschacher *et al.* 2024, Rai *et al.* 2025), showing that the perceptual and acoustic experience of liveness fundamentally alters neural sensitivity to musical rhythm and pleasurable perception of music.

## Supplementary Material

nsag021_Supplementary_Data

## Data Availability

Code and data will be made available upon reasonable request to the corresponding author.
