# Supplementary material for: From lab to concert hall: effects of live performance on neural-acoustic phase-locking and engagement
Source: Soc Cogn Affect Neurosci. 2026 Mar 19;21(1):nsag021. doi: 10.1093/scan/nsag021 (PMC13166874; doi:10.1093/scan/nsag021)

# Supplementary Materials

### Methods

#### Data Acquisition (post-hoc test of loudness)

To confirm that differences in audio levels between live and recorded performances were negligible within-participants, we performed a post-hoc test on the raw audio tracks. We extracted the overall loudness of unnormalized audio (measured in LUFS) for all live and recorded trials using MATLAB’s integratedLoudness function. We entered loudness estimates into a linear mixed effects model (loudness ~ liveness + tempo + (1|sub.idx)) to test for the effect of live versus recorded conditions, while accounting for potential variability in loudness due to the tempo condition. The linear mixed effects model revealed no significant differences in loudness for live versus recorded conditions (β=-0.45, SE=0.54, t=0.83, p=0.41); this suggests that audio levels were indeed very consistent between live and recorded trials. The beta estimate indicates that the expected difference in loudness between live and recorded trials was -0.45 LUFS, which is well below the perceptible threshold.

**Audio Preprocessing (testing effects of normalization)**

We performed post-hoc tests to assess whether normalizing audio levels prior to data analysis suppressed genuine acoustic differences between live and recorded performances. Independent sample non-parametric tests within each tempo condition revealed that the unnormalized audio from live performances did not significantly differ in loudness from the unnormalized audio from speaker recordings (two-sided Wilcoxon rank sum test: fast excerpts: *p=0.33*; slow excerpts: *p=0.24;* Median: live+fast, -36.24 LUFS; recorded+fast, -35.83 LUFS; live+slow, -38.62 LUFS; recorded+slow, -37.74 LUFS). To be sure that audio normalization did not impact our analyses, we repeated our acoustic follow-up analysis using unnormalized audio and found comparable results (i.e., the residual acoustic variability did not significantly explain behavioral or neural outcome variables over and above the effect of the liveness condition).

**EEG Preprocessing (detailed steps)**

EEG data was trimmed to the onset of each excerpt using acoustic triggers presented at the beginning of each excerpt. Channel locations were defined according to the international 10-05 standard with the MNI-BEM template. Data was referenced to the right earlobe (A2) and filtered with a high-cutoff of 58 Hz and a low-cutoff of 0.2 Hz. The low-cutoff was chosen to include the beat rate of the slow excerpts (~50 bpm or .8 Hz). Noisy channels were identified and rejected when the signal was correlated at r<0.5 from the surrounding channels’ random sample consensus (RANSAC) estimate; had a flatline duration of more than 0.5 seconds; or had noise which reached more than 4 standard deviations of the signal. Artifact Subspace Reconstruction (ASR) was used to extrapolate data epochs where the variance was larger than 10 standard deviations from the calibration data [(Chang et al., 2018; Tichko et al., 2022a)](https://www.zotero.org/google-docs/?cNIuDq). After visual inspection of the data to remove additional outlier channels, all rejected channels were interpolated spherically. Independent Component Analysis (ICA) was performed to identify and remove independent spatial components of the data that reflect signal artifacts. The spatial components revealed through ICA were assigned probabilities of belonging to “Brain”, “Muscle”, “Eye”, “Heart”, “Line Noise”, “Channel Noise”, or “Other” classes using the ICLabel function. Components that were assigned the highest probability for the “Brain” class compared to any other class were retained. Components that were assigned a higher probability for any non-brain class were considered for rejection. Components that did not contain characteristic EEG features in the frequency domain (i.e., did not contain an alpha peak between 8-12 Hz or display a 1/f curve) were rejected.

### Figure S1. Post-Excerpt Self-Report Questionnaire

### Participants rated their level of engagement, enjoyment, familiarity, pleasure, focus, investment, distraction, and spontaneity associated with each musical excerpt. The specific wording of each question is detailed in the self-report questionnaire shown below.

**Figure S1. Self-Report Questionnaire**Displayed is a copy of the self-report questionnaire given to participants after every excerpt. Participants answered questions relating to their perceived engagement, enjoyment, familiarity, pleasure, focus, investment, distraction, and spontaneity. Answers were given on a 5-point Likert scale ranging from “Low” to “High” or “Never” to “Always”.


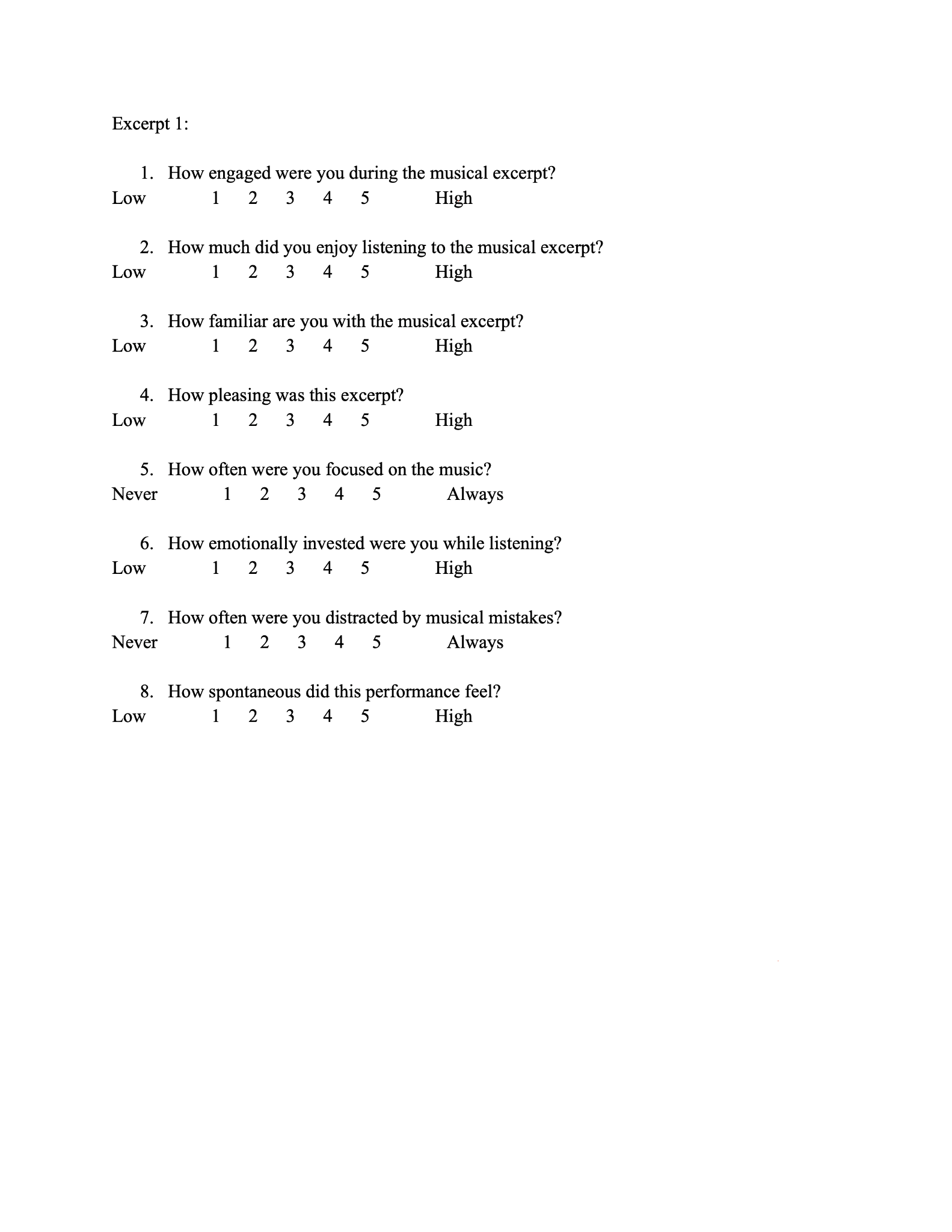


### Figure S2. Variance Explained By Principal Components for Behavioral and Acoustic Analyses

We entered questionnaire scores into a principal components analysis (PCA) to reveal latent sources of variability in the data. In addition, we performed PCA on acoustic features to identify acoustic components that explained variability across live and recorded performances. The choice to retain two principal components in both the behavioral and acoustic PCA analyses rested upon the portion of variance tapering after the second component. Thus, we plot the portion of variance explained for each principal component (scree plots) for the behavioral and acoustic analyses separately. Both plots show that the portion of variance explained by consecutive principal components tapers after the second principal component.

**Figure S2. Portion of Variance Explained for Behavioral and Acoustic Principal Components**(A) A scatter plot of the portion of variance in questionnaire scores explained by each of the eight principal components given by the PCA analysis. We investigated principal components 1 and 2, which explained 48.3% and 16.2% of the total variance respectively. (B) A scatter plot of the portion of variance in acoustic features explained by each of the six principal components given by PCA. We retained principal components 1 and 2, which explained 48.6% and 39.6% of the total variance respectively.

A

B


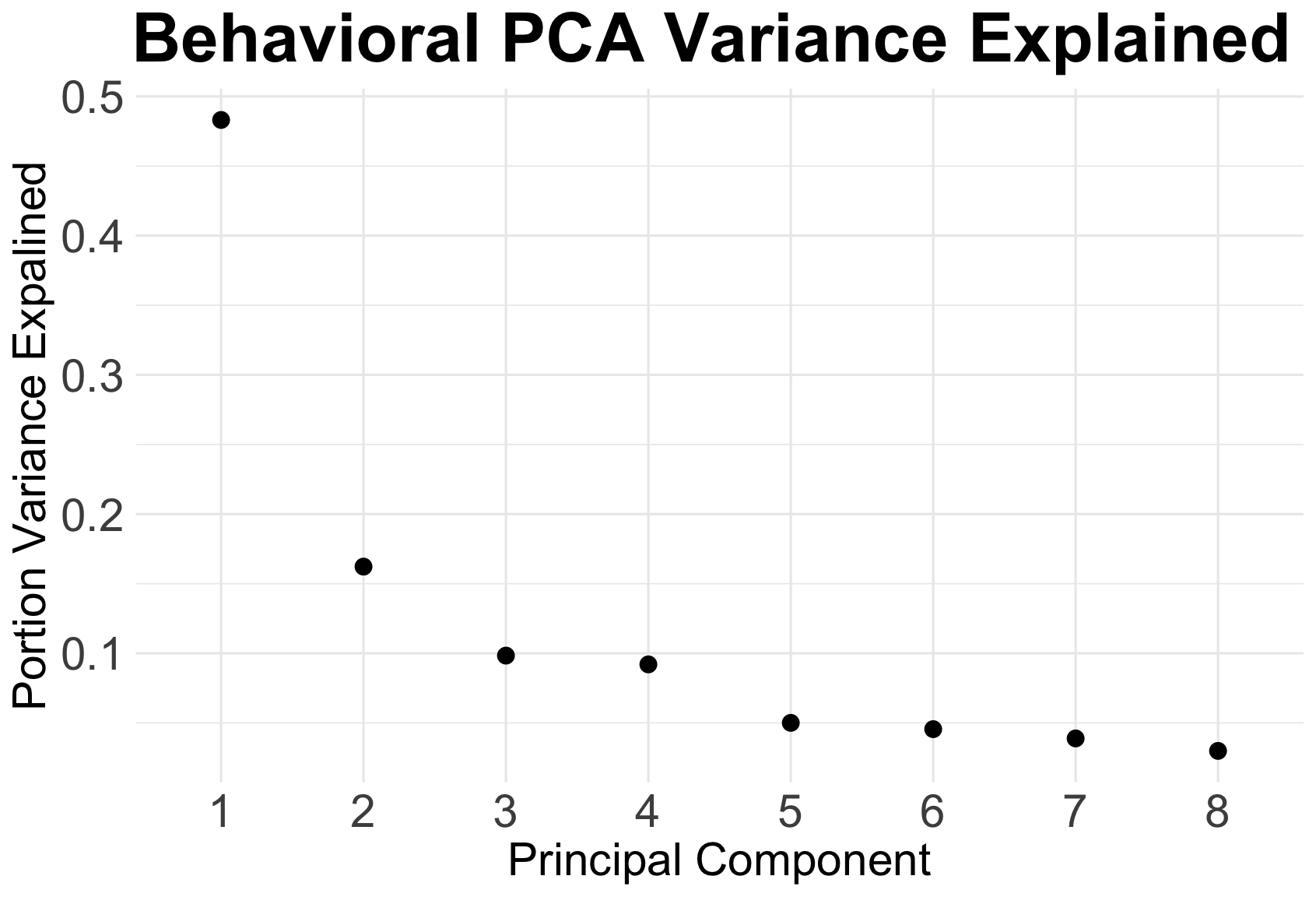

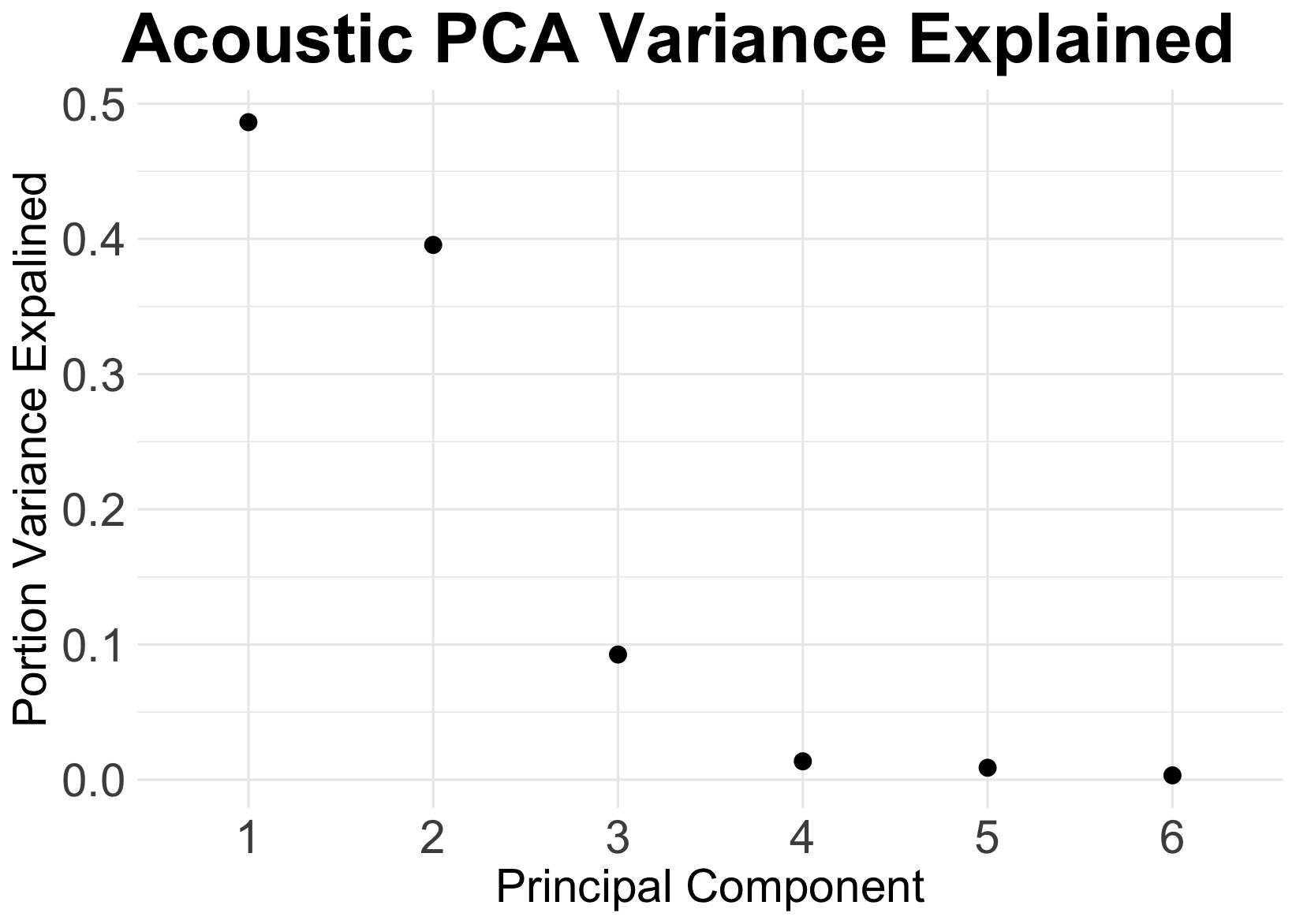


### Figure S3. PCA Loadings For Post-Excerpt Questionnaire Data

Participants gave ratings on engagement, enjoyment, familiarity, pleasure, focus, investment, and distraction, which were subjected to a principal components analysis (PCA). The first principal component scores were referred to as “pleasure-engagement” ratings. Loadings of the individual questionnaire items for this first principal component are shown below.

**Figure S3. Questionnaire PCA Loadings**(A) Circular plot depicting relative loadings of each post-listening questionnaire item for the first two principal components. (B) Item loadings for PC1 and PC2. PC1 was referred to as the “pleasure-engagement” dimension. PC2 was referred to as the “distraction-familiarity” dimension. Loadings with large magnitudes are bolded for each PC.

A.

B.


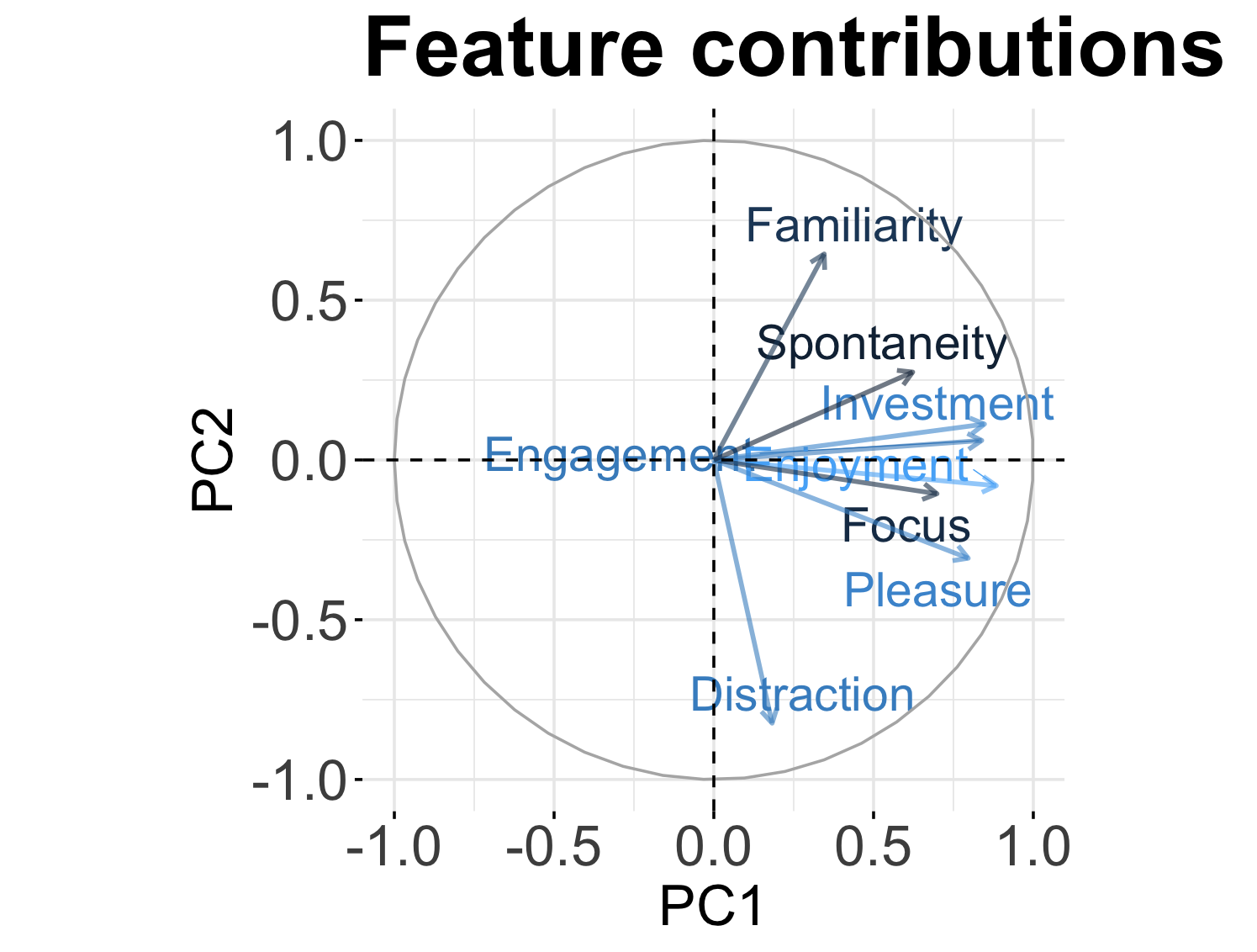

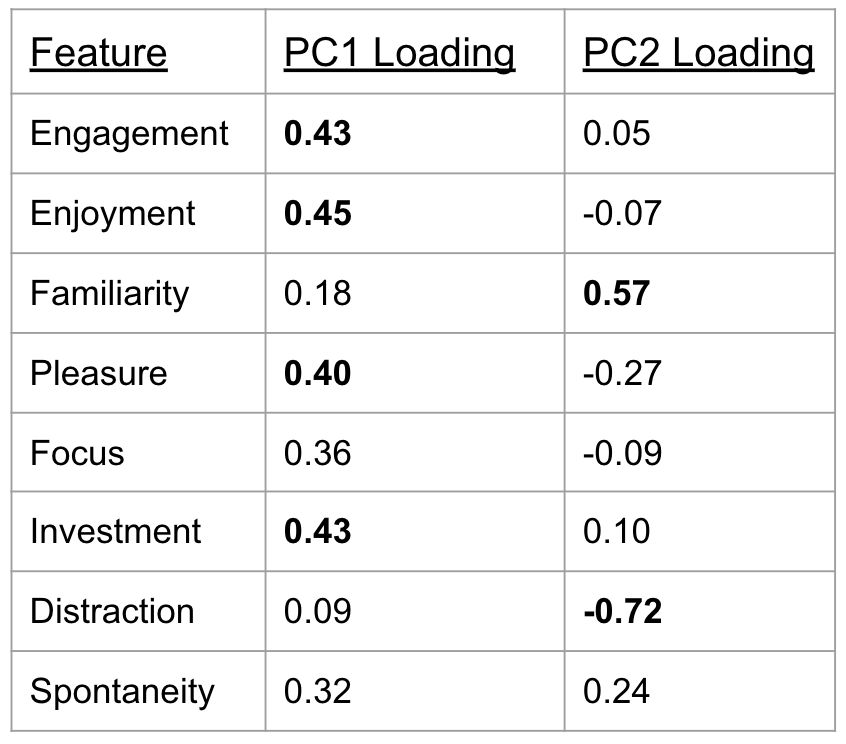


### Figure S4. Acoustic Spectra of Slow and Fast Excerpts

Cochlear-filtered audio was passed through a fast Fourier transform (FFT) to obtain acoustic spectra for slow and fast excerpts. These spectra revealed rhythmic activity in the acoustics across delta and theta frequency ranges. The beat rate and note rates of the slow (~50 bpm) and fast (~125 bpm) excerpts, whose tempi were set prior to data collection, appear as peaks in the amplitude spectra: the beat rate of the slow excerpts (.83 Hz) appears as a peak in the low-delta range and the note rate of the fast excerpts (8.33 Hz) falls within a peak in the upper-theta range.

**Figure S4. Acoustic Spectra of Slow and Fast Excerpts**Acoustic spectra for slow and fast excerpts, averaged across all trials. The beat rate of the slow excerpts (~50 bpm = .83 Hz) appears as a peak in the acoustic spectrum. Similarly, the beat rate and the note rate of the fast excerpts are reflected as separate peaks in the acoustic spectrum in the delta and theta ranges (~125 bpm = 2.083 Hz, 4*125 bpm = 8.3 Hz).


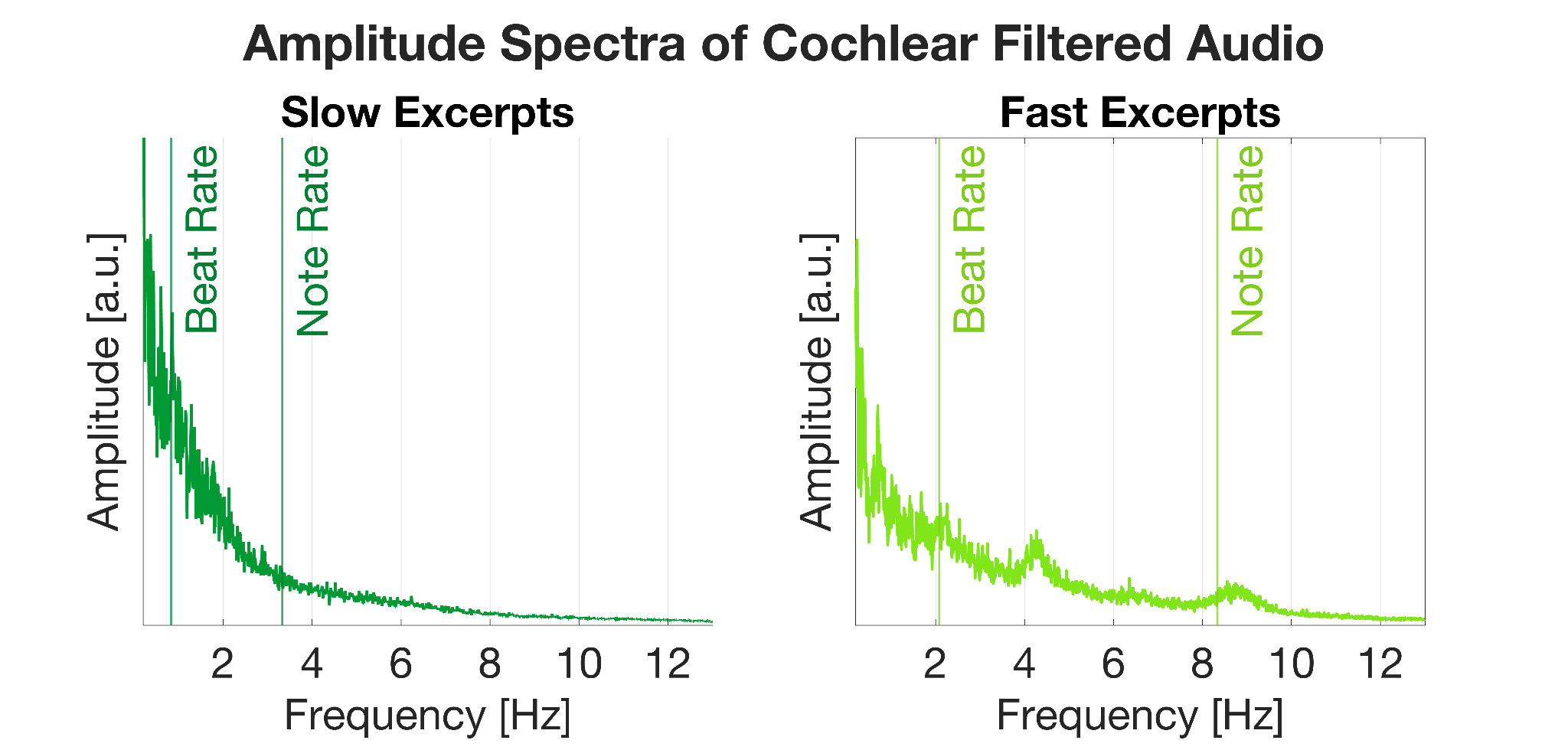


### Table S1. Full Model Estimate For Behavioral Model

**Table S1. Behavioral Generalized Linear Mixed Effects Model Estimates**

The table displays beta estimates, standard errors, z-values, and p-values for each fixed effect from the behavioral model. We used the liveness condition, the tempo condition, and their interaction as fixed-effect regressors on the first principal component of the behavioral ratings.


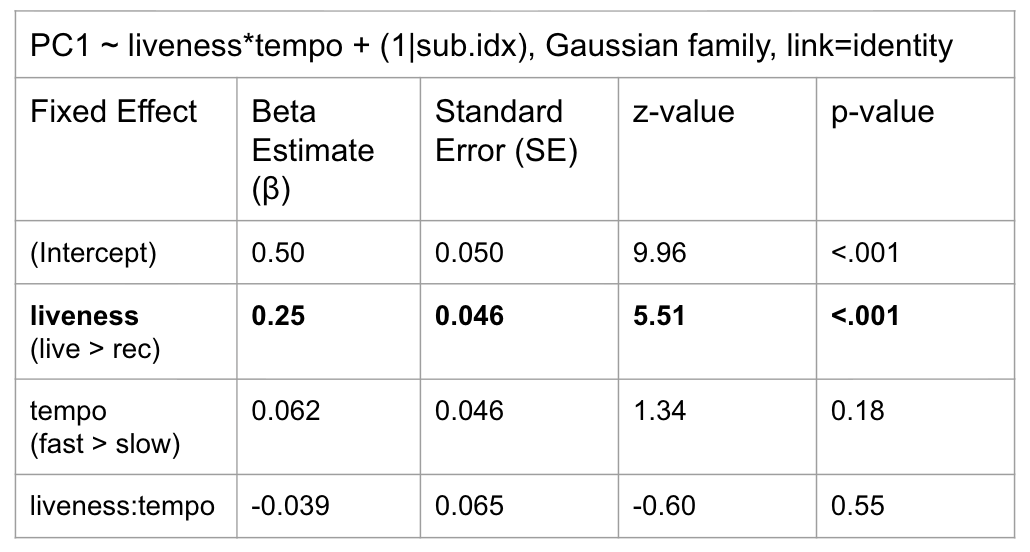


### Table S2. Full Model Estimate For Phase-Locking and Brain-Behavior Models

**Table S2. Phase-locking + Phase-locking to Behavior Generalized Linear Mixed Effects Model Estimates**

(A) The table displays beta estimates, standard errors, z-values, and p-values for each fixed effect from the phase-locking model. We used the liveness condition and the specific musical excerpt as fixed-effect regressors on the average phase-locking value across electrodes over the frequency range of interest (7.6-8.4 Hz) (log-transformed after the link function). (B) The table displays the model estimates for the within-subjects difference in PLV (fixed-effect), which was regressed onto the within-subjects difference in pleasure-engagement ratings (PC1 of the behavioral data).

A

B


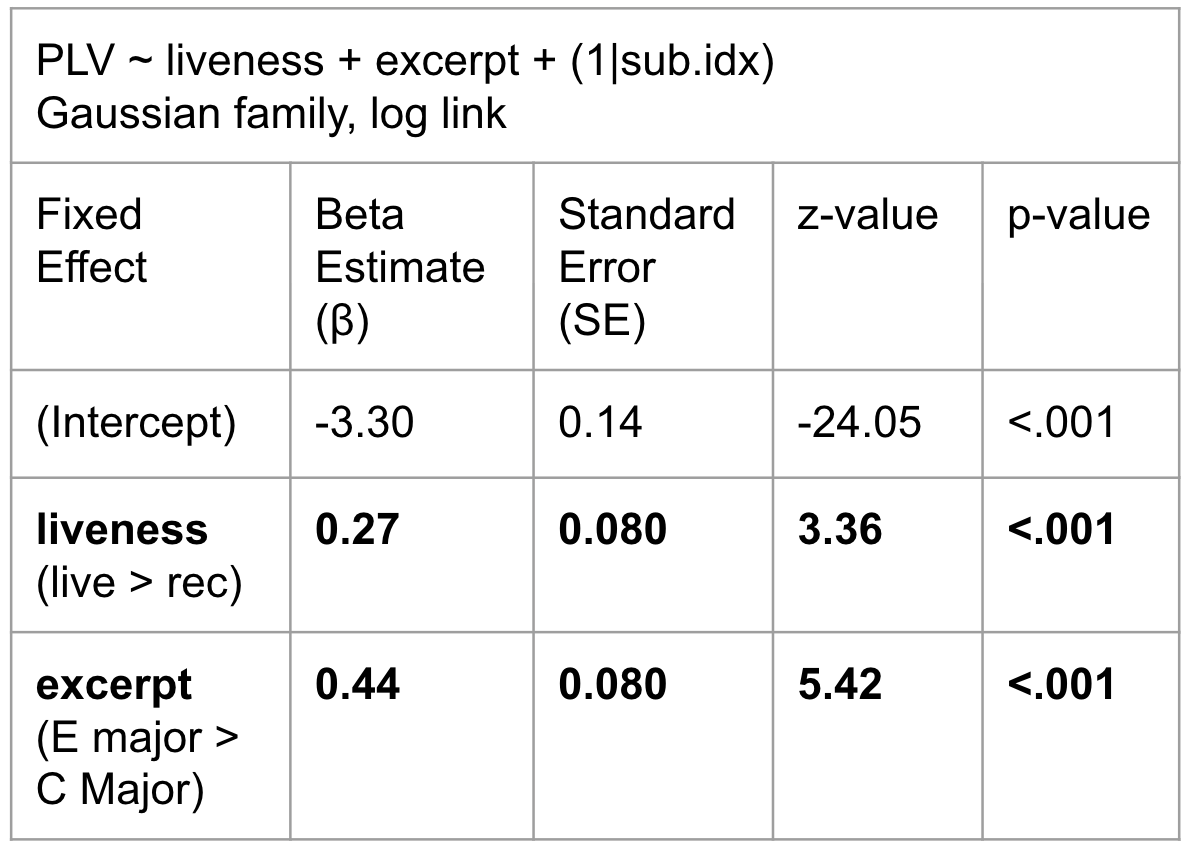

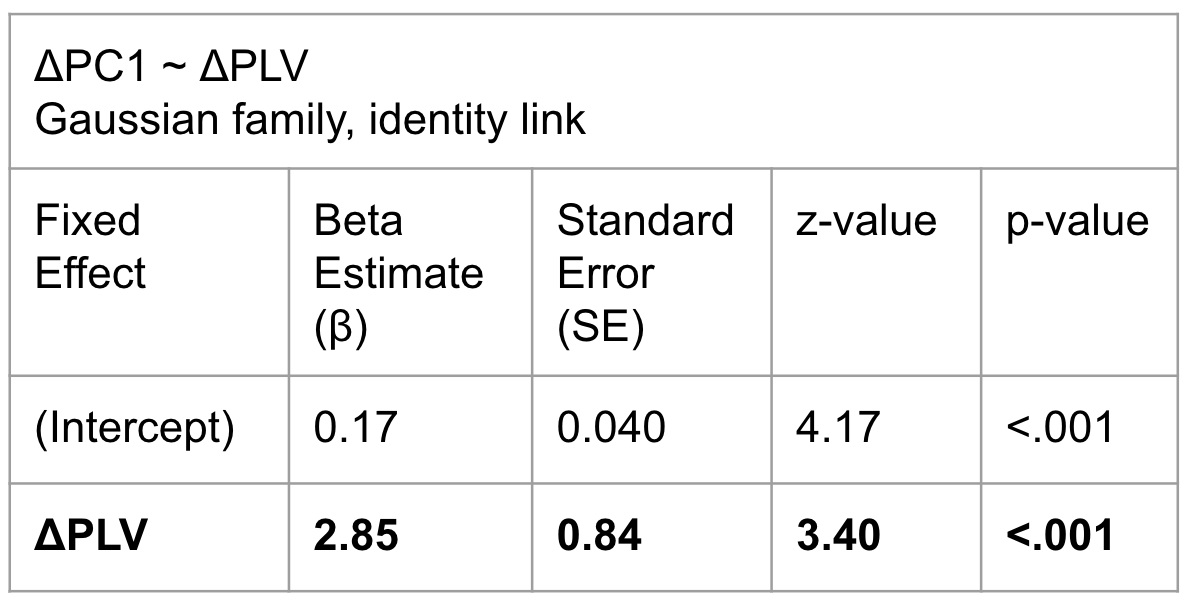


### Table S3. Acoustic Residual Model Estimates

**Table S3. Acoustic Residuals Generalized Linear Mixed Effects Model Estimates**

The tables report beta estimates, standard errors, z-values, and p-values for each fixed effect from the behavioral model (A) and the phase-locking model (B) with the added acoustic residuals regressor. The acoustic residuals represent variance in the first principal component of the acoustics that is not shared with the liveness condition.

A.

B.


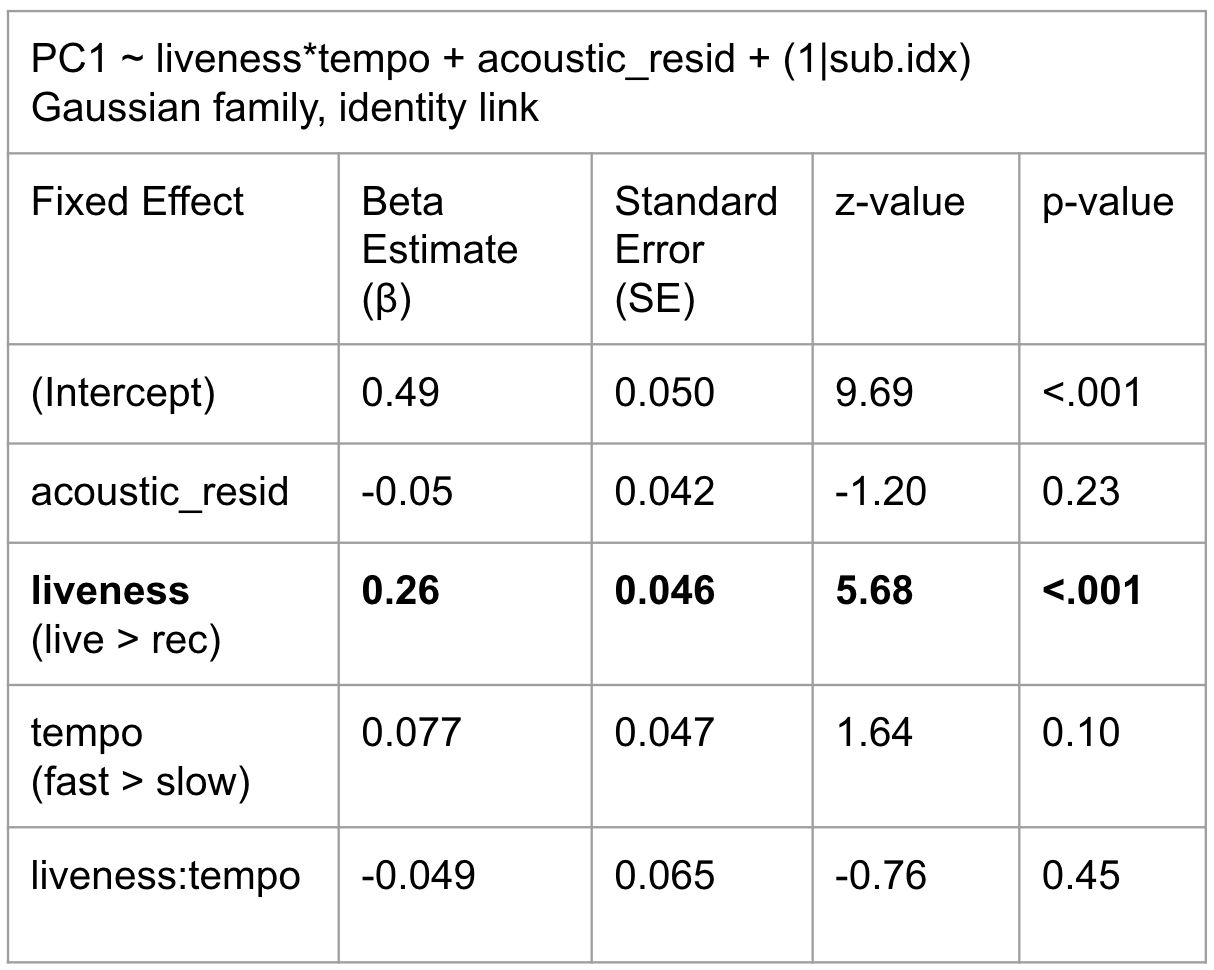

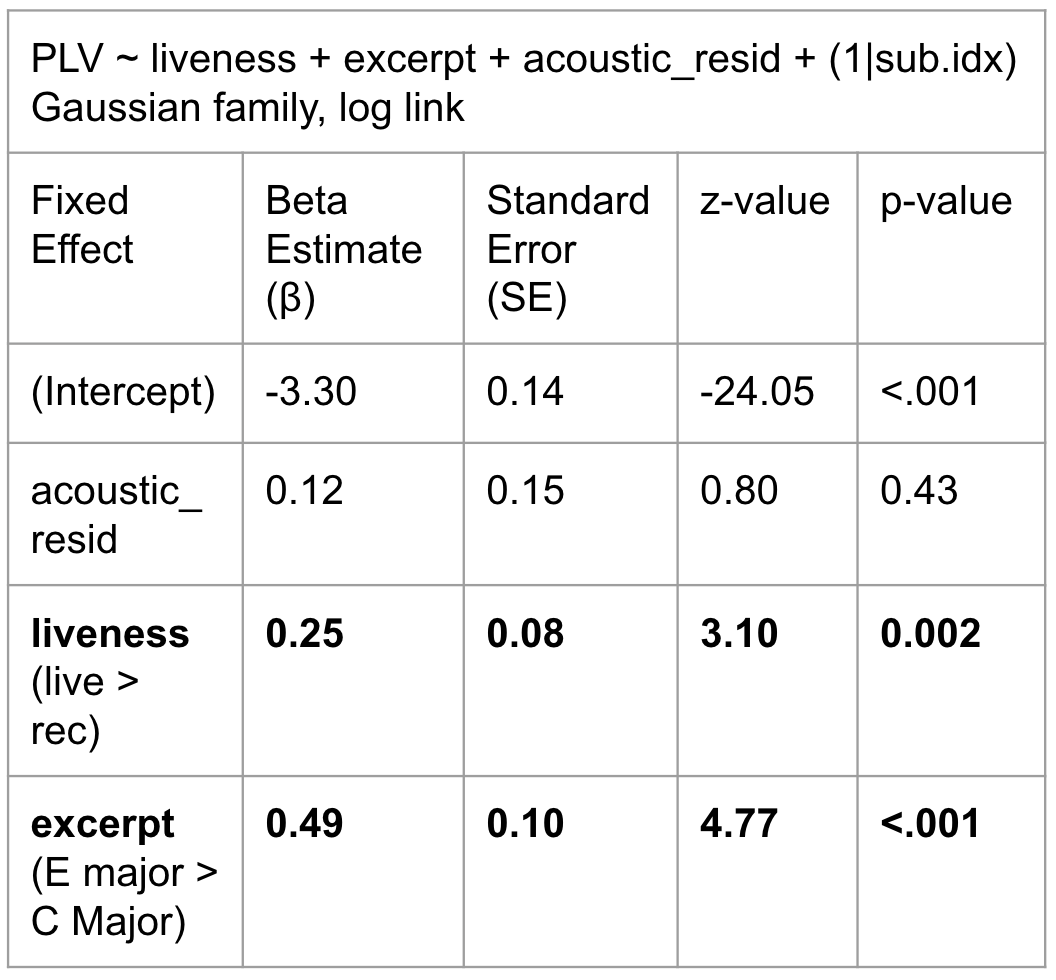

Supplement: nsag021_Supplementary_Data [file nsag021_supplementary_data.docx]
